# Comparison of surgical outcomes between initial trabeculectomy and Ex-PRESS in terms of achieving an intraocular pressure below 15 and 18 mmHg: a retrospective comparative study

**DOI:** 10.1186/s40662-022-00279-1

**Published:** 2022-03-01

**Authors:** Shunsuke Nakakura, Ryo Asaoka

**Affiliations:** 1 Department of Ophthalmology, Saneikai Tsukazaki Hospital, 68-1 Aboshi Waku, Himeji, 671-1227 Japan; 2 Department of Ophthalmology, Seirei Hamamatsu General Hospital, Hamamatsu, Shizuoka Japan; 3 Seirei Christopher University, Hamamatsu, Shizuoka Japan

**Keywords:** Glaucoma, Trabeculectomy, Ex-PRESS, IOP, Goldmann applanation tonometer

## Abstract

**Background:**

To evaluate the postoperative outcomes of initial trabeculectomy (Trab) and Ex-PRESS (EX) in terms of achieving an intraocular pressure (IOP) below 15 and 18 mmHg.

**Methods:**

This study retrospectively analyzed 64 and 54 cases of Trab and EX, respectively, performed by the same surgeon with uniform management from April 2018 to March 2019. Surgical success was defined as 5 < IOP < 15 mmHg (criterion 1) and 5 < IOP < 18 mmHg (criterion 2) without additional glaucoma medication, needling, and bleb reconstruction 2 months after surgery. Survival analysis with Cox regression was performed to identify factors associated with postoperative outcomes.

**Results:**

The Trab and EX groups had an IOP of 22.6 ± 6.2 vs. 21.8 ± 6.0 mmHg before surgery (*P* = 0.507), 12.6 ± 2.6 vs. 14.0 ± 4.4 mmHg (*P* = 0.06) at 6 months, 12.7 ± 2.3 vs. 12.9 ± 2.8 mmHg (*P* = 0.678) at 12 months, 13.3 ± 2.6 vs. 12.6 ± 2.8 mmHg (*P* = 0.260) at 18 months, and 13.2 ± 2.3 vs. 13.6 ± 2.8 mmHg (*P* = 0.444) at 24 months, respectively. The proportion of those with an IOP < 15 mmHg in the Trab and EX groups was 82% vs. 81% at 6 months, 68% vs. 62% at 12 months, 63% vs. 61% at 18 months, and 57% vs. 53% at 24 months, respectively. The log-rank test showed no significant difference between the groups for Criteria 1 (*P* = 0.755) and 2 (*P* = 0.138). The results of the multivariate logistic analysis identified only a high preoperative IOP as a risk factor for surgical failure (odds ratio for Criteria 1: 1.076, *P* = 0.009 and Criteria 2: 1.068, *P* = 0.048).

**Conclusion:**

Postoperative outcomes of Trab and EX suggested similar ability for achieving an IOP below 15 and 18 mmHg without medications and interventions.

**Supplementary Information:**

The online version contains supplementary material available at 10.1186/s40662-022-00279-1.

## Background

Glaucoma remains the second most common cause of blindness worldwide [1]. Estimates have shown that approximately 80 million individuals will be living with glaucoma in the near future, among whom 50% would be unaware of their disease [1]. The only proven approach to address this concern has been the lowering of intraocular pressure (IOP) considering that a 1 mmHg increase in IOP promotes a 10–18% increase in the risk of developing glaucoma [2–4], whereas an IOP reduction of 1 mmHg decreases the risk of progression by about 10% [5]. Recently, clinicians’ preferences have shifted toward minimally invasive glaucoma surgeries, resulting in surgical IOP outcomes of around mid-teens at 1 year [6–10]. Therefore, ophthalmologists perform filtering surgery such as trabeculectomy (Trab) and Ex-PRESS (EX) device implantation are required to produce both high success rates and low IOPs. Xen [11] and Preserflo [12] have recently utilized implant devices like EX, which uses mitomycin C (MMC) and filtering of the aqueous humor into the subconjunctival spaces. Therefore, given the worldwide trend toward minimally invasive surgeries, the surgical outcomes of Trab and EX should be reconsidered to achieve lower IOP. Previous studies comparing Trab and EX have adapted relatively relaxed surgical success criteria for IOP and nonuniform surgical management [13–20]. To determine the actual efficacy of surgery, restarting antiglaucoma medications after surgery should be considered given their influence on success rates.

Here, we evaluated the surgical outcomes of initial Trab and EX performed by a single consultant surgeon. In particular, this study compared the real-world ability of both procedures to achieve an IOP of < 15 and < 18 mmHg without medications and interventions.

## Methods

This retrospective comparative study was approved by the Institutional Review Board of Saneikai Tsukazaki Hospital (IRB No. 211027) and was conducted in accordance with the tenets of the Declaration of Helsinki. Information in the electronic database of the Department of Ophthalmology, Saneikai Tsukazaki Hospital, was collected between April 2018 and March 2019. Initially, all patients (N = 198) who underwent Trab or EX (P50PL) (Alcon Laboratories, Fort Worth, TX, the USA) were assessed (by S.N.) during the study period. Thereafter, patients who underwent simultaneous cataract surgery, those with a history of glaucoma surgery within 3 years, and those who received vitrectomy after Trab or EX were excluded. Similarly, those with postoperative follow-up less than 1 year were excluded. However, patients who skipped their examination for 1 year due to Covid-19 but were re-examined consecutively 15 months after surgery were included. A flowchart for study inclusion is presented in Additional file 1: Fig. S1. In the end, 64 and 54 eyes that underwent initial Trab and initial EX were analyzed, respectively. We investigated patient demographics such as the type of glaucoma, severity of visual field defect, pre- and postoperative IOP, number of antiglaucoma eye drops used, and surgical interventions.

### Surgical techniques and postsurgical management

Trab and EX were performed as similarly as possible, with the only difference being that the Trab surgery was performed with a second flap excision. Initially, a corneal traction suture was placed using a 9–0 silk suture to fix the eye downward. Thereafter, a 7-mm limbal conjunctiva incision and subtenon anesthesia with 2% xylocaine was performed, including epinephrine administration to stop the bleeding. After making spaces under Tenon’s capsule and the conjunctiva for a wider view of the fornix area, coagulation for bleeding was performed, and a 2.5 × 2.5 mm half-thickness quadrangular scleral flap was created. Thereafter, 0.04% MMC was administered under Tenon’s capsule and the conjunctival space for 3 min using neurosurgical pad bemsheets, followed by irrigation with approximately 200 mL of balanced salt solution. This procedure was similarly performed during both surgeries.

In eyes receiving Trab, a second 2.0 × 2.0 mm flap was created and cut off to construct a scleral tunnel. After creating a window anterior to the chamber using V-rance and Kelly punch, peripheral iridectomy was performed. The scleral flap was then sutured using 10–0 nylon for approximately 4 or 5 sutures to adjust the IOP to approximately 10–15 mmHg via palpation. In eyes undergoing EX, a 25-G needle was used to enter the anterior chamber parallel to the iris, after which the EX was implanted. The scleral flap was then sutured using 10–0 nylon for approximately 3 or 4 sutures to adjust the IOP to 10–15 mmHg via palpation.

After the surgery, patients were prescribed 1.5% levofloxacin and 0.1% betamethasone four times per day for approximately 1.5 months with gradual tapering. Laser suture lysis was performed to achieve a target IOP of approximately the low teens 1 week after surgery and 8 to 9 mmHg 2 weeks after surgery. However, laser suture lysis and massage were ineffective for creating blebs corresponding to an IOP at the low teens. Thus, revision surgery using a bleb knife [21] was performed after 1 to 2 months. Those who required needling procedure or bleb revision after 2 months and restarting of glaucoma medication were defined as surgical failures. Around 3 months after surgery, needling was the first treatment of choice for raising IOP after Trab or EX [21], followed by limbal-based bleb revision. Patients who refused surgical repair of the bleb were mostly treated with prostaglandin analog monotherapy.

Oral acetazolamide and topical 1% atropine were administered when choroidal detachment occurred following a decrease in IOP, all of which resolved within 2 months. Patients were scheduled to visit the clinic at postoperative day 1; postoperative weeks 1, 2, and 3; and postoperative months 1, 2, and 3. When no intervention for increasing IOP was required after 3 months, patients were scheduled to visit every 3 months. All surgical procedures, postoperative examinations, laser suture lysis, and interventions (e.g., needling, bleb revisions, and medical prescriptions) were performed by S.N. to control for the effects of surgeon factors (surgical skill, bleb management, strategy, etc.) on surgical outcomes and ensure maximum uniformity in the surgical procedure. Best- corrected visual acuity (BCVA) was evaluated before surgery, 6 months after surgery, 1 year after surgery, and during the last visit. Additional glaucoma surgeries, were also determined via the medical records. IOP was measured using the Goldmann applanation tonometer.

### Statistical analysis

Statistical analyses were performed using JMP version 10.0.0 (SAS Institute Inc., Cary, NC, USA) and statistical program R software (version 3.6.1, http://www.rproject.org/). For the comparison of patient background and frequency of early postoperative complications, Fisher's exact test, Chi-squared test, and Mann-Whitney U test were utilized. IOP and the number of patients using glaucoma eye drops were presented as mean ± standard deviation (SD) and compared using Welch's *t*-test and Mann-Whitney U test, respectively. Baseline BCVA (logMAR) was evaluated via the one-way analysis of variance (ANOVA) test and compared using Welch's *t*-test. Surgical success was defined as 5 < IOP < 15 mmHg (Criterion 1) and 5 < IOP < 18 mmHg (Criterion 2), without any additional glaucoma medication, needling, and bleb reconstruction 2 months after surgery. The surgery was deemed as a failure when the IOP was < 5 mmHg or > 15 mmHg (Criterion 1), and > 18 mmHg (Criterion 2) at two consecutive visits, antiglaucoma medication was restarted, needling revision was performed using MMC [21], or bleb revision and additional glaucoma surgery was conducted at any time after 3 months following surgery.

Survival analysis with Cox regression was performed to identify factors associated with postoperative outcomes. Multivariate logistic regression analysis was conducted using surgery (Trab/EX), age, sex, eye (right/left), glaucoma types, lens status [phakia/intraocular lens (IOL)], preoperative IOP, and the number of preoperative antiglaucoma medications to identify the risk factors for surgical failure. To determine the number of antiglaucoma medications, a fixed combination was calculated as two medications, whereas oral acetazolamide was counted as one medication. *P *values of less than 0.05 were considered statistically significant.

## Results

### Patient demographics

Table 1 summarizes the demographic data of the 64 eyes from the 64 patients in the Trab group and the 54 eyes from the 54 patients in the EX group. The EX group was significantly older (median age, 75 vs. 61 years; *P* < 0.001; Mann-Whitney U test) and had significantly a higher IOL implantation rate (*P* < 0.001; Fisher’s exact test) than the Trab group. No significant differences in sex (*P* = 0.510, Fisher's exact test), treated eye (*P* = 0.430, Fisher's exact test), and glaucoma types (*P* = 0.610; Chi-squared test) were observed between the groups.

**Table 1:** Patient demographics

Characteristics	Trabeculectomy	Ex-PRESS	*P* value
Number at baseline	64	54	
Sex (female), (n, %)	34 (53)	26 (48)	0.510
Treated eye, right (n, %)	37 (57)	33 (61)	0.430
Median age (quantile)	61 (56, 70)	75 (70, 82)	< 0.001
Lens status, intraocular lens (n, %)	20 (31)	54 (100)	< 0.001
Glaucoma type			0.610
POAG (n, %)	46 (72)	35 (65)	
EG (n, %)	7 (10)	11 (20)	
SG (n, %)	8 (12)	5 (9)	
NVG (n, %)	1 (2)	1 (2)	
Pigmentary glaucoma (n, %)	1 (2)	2 (4)	
ACG (n, %)	1 (2)	0 (0)	

*P* values were calculated using Fisher's exact test, Chi-squared test and Mann-Whitney *U* test

*POAG* = primary open-angle glaucoma; *EG* = exfoliation glaucoma; *SG* = secondary glaucoma; *NVG* = neovascular glaucoma; *ACG* = angle closure glaucoma

### IOP and number of antiglaucoma medications

IOP and the number of medications received during follow-up are detailed in Table 2 and 3. IOP (Table 2) is presented together with all antiglaucoma medications and additional interventions. Additional glaucoma surgery comprised μ-hook Trab [22] in the Trab (one case) and EX (one case) groups.

**Table 2:** Intraocular pressure during the follow-up period

IOP (mmHg)	N	Trabeculectomy	N	Ex-PRESS	*P *value
Presurgical	64	22.6 ± 6.2	54	21.8 ± 6.0	0.507
1 W	64	11.9 ± 3.5	54	12.6 ± 6.0	0.447
2 W	64	11.7 ± 3.4	54	12.2 ± 2.9	0.428
1 M	64	12.2 ± 2.7	54	13.0 ± 3.6	0.225
2 M	64	12.9 ± 2.9	54	12.4 ± 2.6	0.363
3 M	64	12.7 ± 2.4	54	12.9 ± 4.3	0.732
6 M	63	12.6 ± 2.6	54	14.0 ± 4.4	0.060
9 M	62	13.1 ± 2.4	53	13.0 ± 3.1	0.947
12 M	64	12.7 ± 2.3	52	12.9 ± 2.8	0.678
15 M	63	13.0 ± 2.7	52	12.8 ± 3.1	0.667
18 M	63	13.3 ± 2.6	51	12.6 ± 2.8	0.260
21 M	62	13.4 ± 1.9	49	13.1 ± 3.4	0.570
24 M	62	13.2 ± 2.3	48	13.6 ± 2.8	0.444
27 M	57	13,3 ± 2.6	48	14.0 ± 4.4	0.349
30 M	36	14.1 ± 2.5	45	13.6 ± 2.9	0.403
33 M	24	14.2 ± 2.8	37	12.7 ± 2.2	0.028
36 M	9	13.1 ± 3.3	19	13.1 ± 2.6	0.971
Final visit	64	13.1 ± 2.4	54	12.7 ± 2.5	0.317

All *P *values were calculated using Welch's *t*-test. *W* = week; *M* = month

**Table 3:** Number of antiglaucoma eye drops during the follow-up period

Number of eye drops	N	Trabeculectomy	N	Ex-PRESS	*P* value
Presurgical	64	3.1 ± 0.9	54	3.3 ± 1.0	0.187
1 W	64	–	54	–	–
2 W	64	–	54	–	–
1 M	64	–	54	–	–
2 M	64	–	54	–	–
3 M	64	0.0 ± 0.1	54	0.0 ± 0.0	0.358
6 M	63	0.0 ± 0.3	54	0.0 ± 0.4	0.790
9 M	62	0.1 ± 0.6	53	0.1 ± 0.6	0.830
12 M	64	0.1 ± 0.6	52	0.1 ± 0.5	0.738
15 M	63	0.2 ± 0.7	52	0.1 ± 0.7	0.487
18 M	63	0.2 ± 0.8	51	0.1 ± 0.6	0.548
21 M	62	0.2 ± 0.8	49	0.3 ± 0.9	1.000
24 M	62	0.2 ± 0.9	48	0.4 ± 1.0	0.410
27 M	57	0.3 ± 0.9	48	0.5 ± 1.1	0.351
30 M	36	0.2 ± 0.8	45	0.4 ± 1.0	0.567
33 M	24	0.6 ± 1.3	37	0.5 ± 1.2	0.584
36 M	9	0.5 ± 1.0	19	0.4 ± 0.9	0.760
Final visit	64	0.2 ± 0.8	54	0.4 ± 1.1	0.351

All *P *values were calculated using the Mann-Whitney U test. *W* = week; *M* = month

After each surgery, IOP decreased significantly at each time point compared with the baseline (*P* < 0.001; paired *t*-test).

The Trab and EX groups had an IOP of 22.6 ± 6.2 vs. 21.8 ± 6.0 mmHg (*P* = 0.507) before surgery, 12.6 ± 2.6 vs. 14.0 ± 4.4 mmHg (*P* = 0.06) after 6 months, 12.7 ± 2.3 vs. 12.9 ± 2.8 mmHg (*P* = 0.678) after 12 months, 13.3 ± 2.6 vs. 12.6 ± 2.8 mmHg (*P* = 0.260) after 18 months, and 13.2 ± 2.3 vs. 13.6 ± 2.8 mmHg (*P* = 0.444) after 24 months, respectively.

No significant differences were found between the groups (all *P* > 0.05) except for that at 33 months, during which the EX group had a significantly lower IOP than the Trab group (14.2 ± 2.8 vs. 12.7 ± 2.2 mmHg; *P* = 0.028). However, the number of patients followed up had decreased by this time (24 vs. 37 in the Trab and EX groups, respectively).

The number of antiglaucoma eye drops decreased significantly from presurgical to each postoperative time point in each group, respectively (all *P* < 0.01; Mann-Whitney U test, Table 3). No significant intergroup differences were noted (all *P* > 0.30).

Presurgical visual field defects measured by the Humphry Field Analyzer (HFA) (24–2 or 30–2 Swedish Interactive Threshold Algorithm Standard test) were only present in 52 and 35 patients in the Trab and EX groups [mean deviation: − 16.03 ± 6.3 vs. − 18.23 ± 6.5 dB for the Trab EX groups, respectively (*P* = 0.117; Welch's *t*-test)]. The remaining patients’ visual field tests could not be analyzed given that they underwent Goldmann perimetry or the HFA 10–2 program.

### Visual acuity

The EX group had significantly worse baseline BCVA than the Trab group (0.29 ± 0.28 vs. 0.17 ± 0.18 logMAR, respectively; *P* = 0.009). However, there was no significant change in BCVA between each group during follow-up (before surgery, after 6 and 12 months of surgery, and during the final visit) (*P* = 0.863 and 0.494 in the Trab and EX groups, respectively; one-way ANOVA).

### Complications within 1 month after surgery

No significant difference in the presence of a shallow anterior chamber, choroidal detachment, Seidel treated by a soft contact lens, hyphema, iris inlaying for scleral window or device, and vitreous extrusion in the anterior chamber were observed between the groups (all *P* > 0.20, Fisher's exact test, Table 4).

**Table 4:** Complications within 1 month after surgery

Complications within 1 month after surgery	Trabeculectomy	Ex-PRESS	*P *value
Shallow anterior chamber	5 (8%)	3 (6%)	0.457
Choroidal detachment	5 (8%)	4 (7%)	0.606
Seidel treated by a soft contact lens	5 (8%)	3 (6%)	0.457
Hyphema	5 (8%)	2 (4%)	0.295
Iris inlaying for scleral window or device	0 (%)	1 (2%)	0.457
Vitreous extrusion in anterior chamber	1 (2%)	0 (0%)	0.542

All *P* values were calculated using Fisher's exact test

### Survival analysis results

The median follow-up period (quantile) was 743 days (231–844 days) and 805 days (217–976 days) in the Trab and EX groups, respectively (*P* = 0.592, Welch's *t*-test). In our study, 58 events occurred in Criteria 1 and 43 events occurred in Criteria 2.

Criterion 1. 5 < IOP < 15 mmHg without any additional glaucoma medication or intervention.

**Fig. 1:**
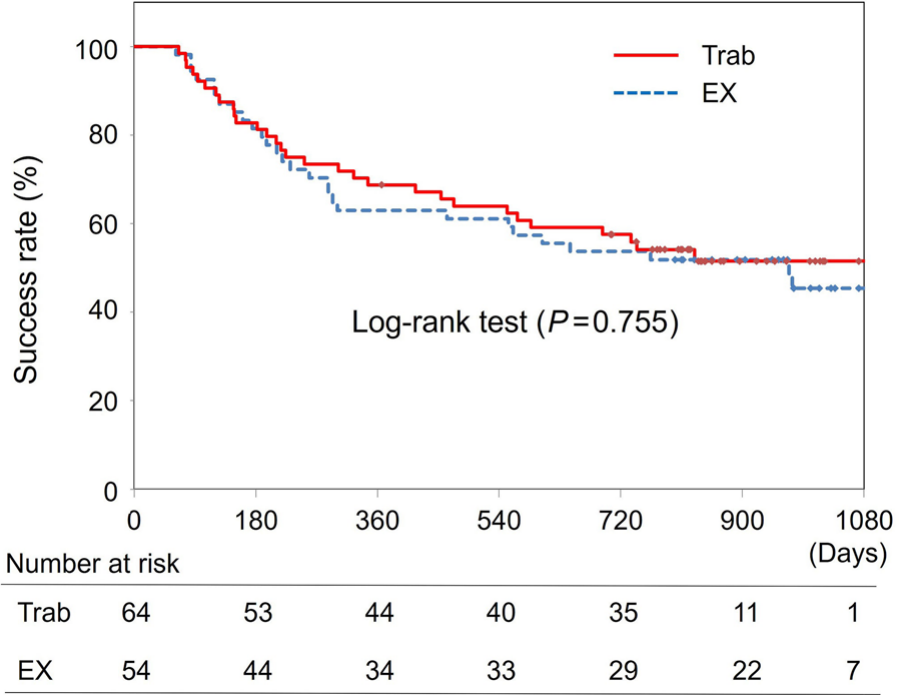
Kaplan-Meier life table with log-rank test for Criterion 1. Criterion 1: 5 < IOP < 15 mmHg without any additional glaucoma medication or interventions. The Kaplan–Meier life table with log-rank test showed no significant difference between the groups (*P* = 0.755). Trab, trabeculectomy; EX, Ex-PRESS

The Kaplan-Meier life table with log-rank test (Fig. 1) showed no significant difference between the groups (*P* = 0.755). Survival rates in the Trab and EX groups were 82% vs. 81% at 6 months, 68% vs. 62% at 12 months, 63% vs. 61% at 18 months, and 57% vs. 53% at 24 months, respectively. Multivariate logistic regression analysis identified preoperative IOP (*P* = 0.009) but not surgery (Trab/EX, *P* = 0.922) as a risk factor for surgical failure with an odds ratio of 1.076 [95% confidence interval (CI): 1.018 to 1.138].

Criterion 2. 5 < IOP < 18 mmHg without any additional glaucoma medication or intervention.

**Fig. 2:**
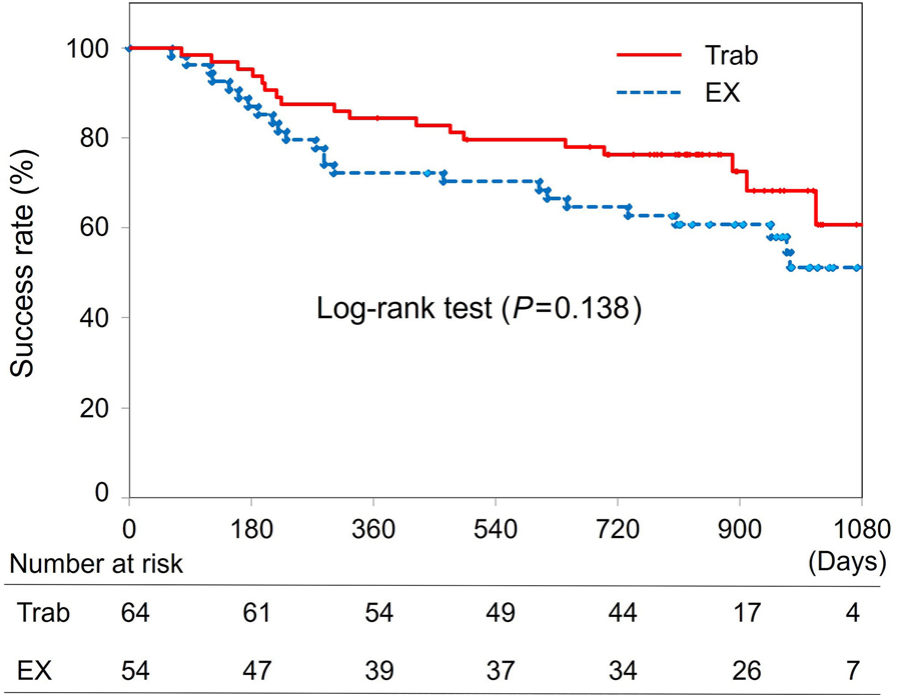
Kaplan-Meier life table with log-rank test for Criterion 2. Criterion 2: 5 < IOP < 18 mmHg without any additional glaucoma medication or intervention. The Kaplan-Meier life table with log-rank test showed no significant difference between the groups (*P* = 0.138). Trab, trabeculectomy; EX, Ex-PRESS

The Kaplan-Meier life table with log-rank test (Fig. 2) showed no significant difference between the groups (*P* = 0.138). Survival rates in the Trab and EX groups were 95% vs. 87% at 6 months, 84% vs. 72% at 12 months, 79% vs. 70% at 18 months, and 72% vs. 64% at 24 months, respectively. Multivariate logistic regression analysis identified preoperative IOP (*P* = 0.048) but not surgery (Trab/EX, *P* = 0.701) as a risk factor for surgical failure, with an odds ratio of 1.068 (95% CI: 1.000 to 1.140).

## Discussion

The present study showed the real-world surgical outcomes of Trab and EX under uniform management using two strict criteria. Few studies that are available have compared Trab and EX over a follow-up duration of more than 1 year (Table 5) [13–20]. Moreover, studies varied in terms of the primary success criteria utilized, with almost all using 5 < IOP < 18 or < 20 mmHg with medications as their primary success criteria, which were less strict than our second criteria. However, de Jong et al., who used criteria similar to us (i.e., 15 and 18 mmHg for Criteria 1 and 2), showed that EX was superior to Trab under both criteria during all follow-up time points, which was contrary to our findings. Similar to this study, surgical procedures by de Jong et al. were completed by a single surgeon; however, they did not include patients with secondary glaucoma, neovascular glaucoma, and angle closure glaucoma, which may have caused the discrepancy in results.

**Table 5:** Summary of efficacy outcomes comparing Trab and EX over a follow-up duration of more than 1 year

Author (year)	Study design	Number of eyes Trab/EX	Primary surgical success definition	Secondary surgical success definition	Primary success rate at 1 year	Primary success rate at 2 years	Secondary success rate at 1 year	Secondary success rate at 2 years
Gonzalez-Rodriguez (2016) [13]	RCT	31/32	5 ≤ IOP ≤ 18 mmHg, a ≥ 20% reduction, no medications	5 ≤ IOP ≤ 18 mmHg, a ≥ 20% reduction, with or without medications	NA	42% vs. 43% (*P* = 0.78)	NA	76% vs. 59% (*P* = 0.20)
Netland (2014) [14]	RCT	61/59	5 ≤ IOP ≤ 18 mmHg, with or without medications and without additional glaucoma surgeries	5 ≤ IOP ≤ 15 mmHg, with or without medications and without additional glaucoma surgeries	87% vs. 90%	79% vs. 83%	70% vs. 73%	56% vs. 58%
Dahan (2012) [15]	RCT	15/15	5 < IOP < 18 mmHg, without medications	5 < IOP < 18 mmHg, with or without medications	NA	NA	NA	NA
de Jong L (2011) [16]	RCT	39/39	4< IOP ≤ 18 mmHg, no medications	4 < IOP ≤ 15 mmHg, no medications	61.5% vs. 86.8% (*P* = 0.01)	51.3% vs. 76.3% (*P* = 0.02)	51.3% vs. 80.0% (*P* = 0.01)	48.7% vs. 71.1% (*P* = 0.046)
Wang (2017) [17]	Prosp	24/24	5 ≤ IOP ≤ 21 mmHg, without surgical interventions	NA	87.5% vs. 95.8% (*P* = 0.289)	NA	NA	NA
Tojo (2018) [18]	Retro	39/69	IOP ≤ 15 mmHg or a ≥ 20% reduction, with or without medications	IOP ≤ 21 mmHg or a ≥ 20% reduction, with or without medications	NA	NA	NA	NA
Liu (2015) [19]	Retro	17/16	5 < IOP < 18 mmHg, no medications	5 < IOP < 18 mmHg, with or without medications	47% vs. 43% (*P* > 0.05)	NA	76.5% vs. 75.0% (*P* > 0.05)	NA
Moisseiev (2015) [20]	Retro	61/39	≤ 20 mmHg or a ≥ 20% reduction, no medications	≤ 20 mmHg or a ≥ 20% reduction, with or without medications	62.3% vs. 66.6%	NA	86.9% vs. 84.6%	NA
Present study	Retro	64/54	5 < IOP < 15 mmHg without medications and interventions	5 < IOP < 18 mmHg without medications and interventions	68% vs. 62%	57% vs. 53%	84% vs. 72%	72% vs. 64%

*Trab* = trabeculectomy; *EX* = Ex-PRESS; *RCT* = randomized control trial; *IOP* = intraocular pressure; *NA* = not applicable; *Prosp* = prospective; *Retro* = retrospective

Most studies showed that Trab and EX had similar IOP lowing efficacy. Notably, three studies utilized primary criteria similar to that of our Criterion 2 [13, 15, 19]. A randomized control study by Gonzalez-Rodriguez JM et al. [13] reported primary success rates of 42% and 43% at 2 years following Trab and EX, respectively (≤ 18 mmHg, i.e., our Criterion 2, *P* = 0.78). Meanwhile, Dahan et al.’s study showed no concrete success rate at any year [15]. In contrast, a retrospective study by Liu et al. reported a primary success rate of 47% and 43% after Trab and EX at 1 year, respectively (< 18 mmHg, i.e., our Criterion 2, *P* > 0.05) [19]. Although the aforementioned study considered Trab and EX to have similar success rates for achieving an IOP of under 18 mmHg without any medication or intervention, their success rates were slightly lower than those presented herein (72% vs. 64% for the Trab and EX groups, respectively). This study identified preoperative IOP as a significant risk factor for surgical failure in both surgical procedures. The Collaborative Bleb-related Infection Incidence and Treatment Study that utilized a large data set also reported preoperative lens status and preoperative higher IOP as significant risk factors for Trab surgical failure [23]. Moreover, the Advanced Glaucoma Intervention Study (AGIS) study found that younger age and higher IOP were significant risk factors for Trab success [24]. Meanwhile, only a handful of studies have identified significant risk factors for EX failure. Only Mariotti et al. had reported risk factors for EX failure, which included diabetes, non-Caucasian ethnicity, and previous glaucoma surgery [25]. However, they did not include preoperative IOP and number of antiglaucoma eye drops before surgery as potential risk factors. Therefore, our study presents new findings for estimating the surgical success of EX. A recent retrospective study [26] reported surgical success rates of 46 and 58% at 1 year and 34 and 49% at 2 years following Xen and Preserflo, respectively, with their criterion for surgical success being ≤ 18 mmHg without medications and additional interventions. Wagner et al., who retrospectively compared the surgical outcomes between Trab and Xen [27], reported 1-year success rates (IOP ≤ 18 mmHg without medications and additional interventions, consistent with our Criterion 2) of 65.5% and 58.5% follow Trab and Xen, respectively. Meanwhile, a prospective study by Beckers et al. on the outcomes of Preserflo after a follow-up period of 2 years [28] reported a complete success rate of 54.3% at 1 year and 54.3% at 2 years (calculated by us) using the criterion IOP < 15 mmHg and ≥ 6 mmHg without any medication and intervention. They also reported a complete success rate of 58.0% at 1 year and 59.2% at 2 years (calculated by us) using the criterion IOP < 18 mmHg and ≥ 6 mmHg without any medication and intervention [28]. Recently, Stoner et al. retrospectively compared Xen and EX. They defined success as an IOP of 6–15 mmHg or 6–18 mmHg, without reoperation for uncontrolled glaucoma, loss of light perception, or the use of glaucoma medication at 1 year. The results of Xen vs. EX were 15.6% vs. 55.6% and 17.8% vs. 55.6%, respectively. Therefore, they concluded that Xen was inferior to EX for complete success [29]. Further studies are needed to determine whether all filtering surgeries using MMC for controlling IOP via laser suture lysis through a scleral flap are superior to single insertion Xen and Preserflo.

The present study has several limitations that are worth noting. First, given that our study was retrospective in nature and utilized real-world data, differences in patient backgrounds could not be adjusted for. Accordingly, the EX group were older, with a higher IOL implantation rate, and worse visual acuity. This bias may be attributed to our knowledge that EX promoted rapid visual acuity recovery to baseline after the surgery [14] and that inserting EX is safer in patients with IOL to prevent the device from touching the iris. The second limitation concerns the cost-effectiveness of IOP reduction. Several problems related to costs have remained for EX [30, 31], which should be discussed in studies regarding recent micro incision glaucoma devices in the near future. Third, the recent Japanese national survey showed that EX had significantly higher reoperation rates than Trab (adjusted hazard ratio, 1.72; *P* < 0.001) [31]. In addition, we excised the second flap via Trab. Dada et al. recently reported that deep sclerectomy—an IOP-lowering procedure, like our method—was superior to single-flap Trab based on a randomized control study (12.5 ± 1.67 vs. 13.4 ± 1.83 mmHg at 12 months, *P* = 0.04) [32]. Therefore, further long-term studies are warranted.

## Conclusion

Our study revealed that Trab and EX exhibited similar ability for achieving an IOP below 15 and below 18 mmHg without medications and interventions. Future prospective studies should determine the proper indications for Trab, EX, and minimally invasive glaucoma surgery to control IOP for glaucoma management.

## Supplementary Information

**Additional file 1: Figure S1.** Flow chart for patient inclusion and exclusion. Trab, trabeculectomy; EX, Ex-PRESS.

## Data Availability

The datasets used in the current study are available from the corresponding author (SN) upon reasonable request.

## Abbreviations

BCVA: Best-corrected visual acuity
EG: Exfoliation glaucoma
EX: Ex-PRESS
IOP: Intraocular pressure
NVG: Neovascular glaucoma
POAG: Primary open-angle glaucoma
SD: Standard deviation
SG: Secondary glaucoma
Trab: Trabeculectomy
