# Supplementary figures and images for: Comparison of surgical outcomes between initial trabeculectomy and Ex-PRESS in terms of achieving an intraocular pressure below 15 and 18 mmHg: a retrospective comparative study
Source: Eye Vis (Lond). 2022 Mar 1;9:9. doi: 10.1186/s40662-022-00279-1 (PMC8885135; doi:10.1186/s40662-022-00279-1)

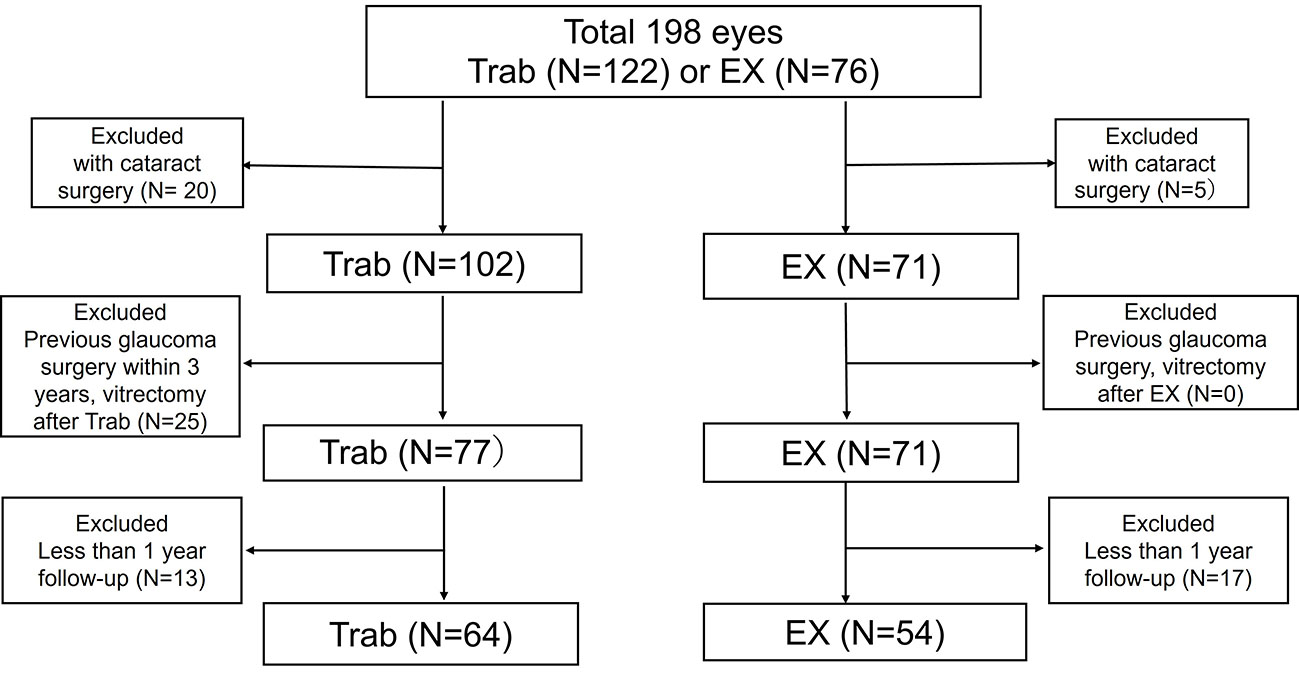

Supplement: Supplementary file 1 — Additional file 1: Figure S1. Flow chart for patient inclusion and exclusion. Trab, trabeculectomy; EX, Ex-PRESS. [file 40662_2022_279_MOESM1_ESM.jpg]
